# Morphometrical analysis of transbronchial cryobiopsies

**DOI:** 10.1186/1746-1596-6-53

**Published:** 2011-06-16

**Authors:** Sergej Griff, Wim Ammenwerth, Nicolas Schönfeld, Torsten T Bauer, Thomas Mairinger, Torsten-Gerriet Blum, Jens Kollmeier, Wolfram Grüning

**Affiliations:** 1 Institute of Pathology, HELIOS Klinikum Emil von Behring, Berlin, Germany; 2 Department of Pneumology, Lungenklinik Heckeshorn, HELIOS Klinikum Emil von Behring, Berlin, Germany

## Abstract

The recent introduction of bronchoscopically recovered cryobiopsy of lung tissue has opened up new possibilities in the diagnosis of neoplastic and non-neoplastic lung diseases in various aspects. Most notably the morphological diagnosis of peripheral lung biopsies promises to achieve a better yield with a high quality of specimens. To better understand this phenomenon, its diagnostic options and perspectives, this study morphometrically compares 15 cryobiopsies and 18 transbronchial forceps biopsies of peripheral lung tissue a priori without considering clinical hit ratio or integration of results in the clinical diagnostic processing. Cryotechnically harvested specimens were significantly larger (mean: 17.1 ± 10.7 mm^2 ^versus 3.8 ± 4.0 mm^2^) and contained alveolar tissue more often. If present, the alveolar part in cryobiopsies exceeded the one of forceps biopsies. The alveolar tissue of crybiopsy specimens did not show any artefacts. Based on these results cryotechnique seems to open up new perspectives in bronchoscopic diagnosis of lung disease.

## Background

The diagnostic yield of transbronchial lung biopsy (TBB) by forceps is a function of biopsy quality defined by specimen size and preservation of tissue architecture. In addition, artifacts may considerably affect the interpretation of the tissue obtained.

The tissue sample delivered in clinical routine usually consists of one or more lung pieces averaging 1 to 2 mm in size [1-4]. It is difficult to report the diagnostic accuracy of TBB, because they are taken for various indications. The majority of large case series report a diagnostic accuracy of 50% to 70% depending on the indication, size and location of the lesion [1,3,5-10]. Peripheral tumour lesions can be diagnosed in up to 57% of patients [9,11]. In diffuse lung diseases, the overall efficacy is probably lower, whereas the technique seems to be highly efficient in sarcoidosis and cryptogenic organising pneumonia. The results in usual interstitial pneumonia, pneumoconiosis or pulmonary histiocytosis X are poor [1,4,10,12]. This large variation is due to the different importance of alveolar tissue which is usually underrepresented in TBB. Moreover, information about distribution of the pathologic pattern throughout the lungs can principally not be provided by TBB.

Cryosurgical techniques have been used in the airways as early as 1968 [13]. The cryotechnique was mainly used for palliative treatment of obstructing endobronchial tumors [14-16]. Cryotechniques use very low temperatures induced by rapid expansion of gas released at high flow (Joule-Thompson effect) and leads to adhesion of the specimen to the probe. Such pieces of tissue can be extracted with the freeze-thaw cycle without increasing the danger of life threatening complications [17,18].

With the implementation of flexible probes for the diagnostic work-up of patients with endobronchial tumor lesions, cryobiopsy was introduced recently on a routine basis and found to be also safe in a routine diagnostic setting [17,19]. The biopsies obtained in these experiments were reported to be larger and diagnostically more valuable. An animal study supports these hypothesis by showing that the preservation of samples and sample size can be improved by cryotechnique[17,18]. Similar results have been shown from central cryobiopsies in a study on efficacy of cryobiospies in cancer patients [20].

The aim of our study was to evaluate cryotechnique for peripheral transbronchial lung biopsies with the focus on sample adequacy for diagnostic purposes with respect to sample size and proportion of alveolar tissue retrieved.

## Methods

This is a prospective case series of 15 patients, who underwent flexible bronchoscopy including transbronchial cryobiopsies. A series of 18 patients undergoing conventional peripheral transbronchial biopsies by forceps were selected as the control group. Patient characteristics (localization, clinical indication) are listed in tables 1 and 2.

**Table 1:** Measurements of the cryobiopsies:

Specimen's number	**Whole area [mm**^**2**^**]**	Alveolar part within biopsy	**Area of alveolar part [mm**^**2**^**]**	Artifacts of alveolar part	Localisation	Clinical indication
1	31.885	No			OL right	Fibrosis

2	0.633	No			ML	Infiltration

3	4.913	Yes	1.799	None	ML	Infiltration

4	8.813	Yes	5.379	None	OL left	Infiltration

5	28.699	Yes	22.689	None	Lingula	Fibrosis

6	9.125	No			OL right	Fibrosis

7	29.353	Yes	13.303	None	OL left	CUP

8	21.299	Yes	7.035	None	OL right	Fibrosis

9	16.259	No			Ul right	Infiltration

10	14.613	Yes	13.204	None	OL right	Infiltration

11	13.815	Yes	0.449	None	ML	Infiltration

12	31.7999	Yes	25.155	None	UL left	Infiltration

13	28.202	Yes	24.979	None	ML	ILD

14	6.304	Yes	5.684	None	UL left	ILD

15	10.440	Yes	7.503	None	UL left	Infiltration

**Table 2:** Measurements of the forceps biopsies:

Specimen's number	**Whole area of the largest specimen [mm**^**2**^**]**	Alveolar part within biopsy	**Area of alveolar part [mm**^**2**^**]**	Artifacts of alleolar part	Localisation	Clinical indication
1	2.333	Yes	1.058	Severe	UL right	Fibrosis

2	2.093	Yes	0.982	Mild	OL right	Fibrosis

3	1.007	No			ML	Fibrosis

4	1.384	No			ML	Fibrosis

5	4.344	Yes	1.612	Mild	Ul left	ILD

6	1.714	Yes	1.105	Mild	Lingula	Infiltration

7	3.684	Yes	1.195	Moderate	OL right	Infiltration

8	5.142	Yes	2.674	Moderate	OL right	Infiltration

9	2.688	Yes	0.430	Severe	OL right	Infiltration

10	11.828	No			OL right	Peripheral lesion

11	2.424	Yes	1.511	Moderate	UL left	Peripheral lesion

12	0.572	No			ML	Infiltration

13	2.806	Yes	2.372	Severe	UL right	Fibrosis

14	1.422	No			OL right	Infiltration

15	6.817	Yes	5.972	moderate	UL right	Infiltration

16	2.043	No			UL right	Infiltration

17	15.713	No			UL right	Infiltration

18	0.366	No			Lingula	Fibrosis

Cryobiopsies and conventional transbronchial biopsies were obtained during flexible bronchoscopy with sedation and local anaesthesia using a flexible bronchoscope (1T 160 and 1T 180, Olympus Corp. Tokyo Japan). The cryoprobe or forceps were introduced into the selected area under fluoroscopic guidance. For all samples, a distance of approximately 10-20 mm from the thoracic wall was considered optimal. Once brought into position, the probe was cooled for approximately five seconds and then retracted with the frozen lung tissue being attached on the probe's tip. The frozen specimen was thawed in saline and fixed in 4% buffered formalin. All specimens won by TBB were stored in Formalin only and the forceps size used was 1.8 mm (Boston Scientific Radial Jaw™ Natick, MA USA).

For this study a signed informed consent of all patients was obtained. The cryotechnique is a technology authorized by the German medicinal products act and was applied depending on the individual medical indication. Since there was no patient randomization and no specimens beyond the medical indication were harvested, formal approval of an ethics committee was not obtained.

Only one cryobiopsy was taken, whereas the number of conventional TBB varied from 1 to 4, depending on the investigator. All biopsies were processed conventionally by serial sectioning of at least 12 H&E stained section steps to avoid incomplete sectioning of particles. Subsequently the biopsies were rated regarding quality and quantity by two experienced lung pathologists (SG & TM), rendering a consensus about biopsy quality as well as presence and amount of artefacts (Tables 1 and 2).

**Figure 1:**
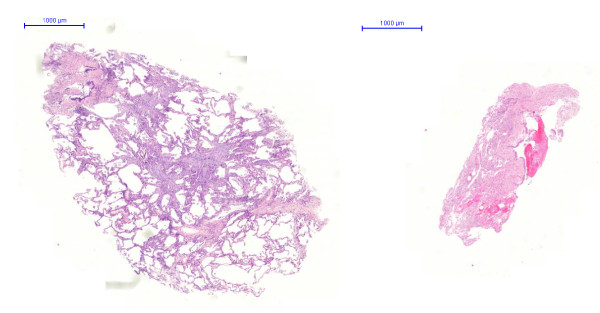
**left: Cryobiopsy, right: Conventional forceps biopsy**.

Hematoxylin-eosine slides were scanned by a ZEISS-MIRAX Midi Slide scanning system using the Mirax Viewer Image Software Ver[1,6]. (Zeiss Microimaging, Oberkochen, Germany and 3DTech, Budapest, Hungary) (Figure 1). The total area of the biopsy specimens and the area of the alveolar part were measured by interactive circling of the biopsy section and its alveolar part. In each conventional TBB, all tissue samples were measured, but only the largest in size was included in the subsequent analysis (see discussion). All areas were calculated automatically and expressed in μm^2^.

## Statistics

All data were analyzed and processed using statistical software (Statistical Package for Social Sciences, Version 14.0; SPSS, Chicago, IL, USA) on a Windows XP operating system (Microsoft; Redmond, WA, USA). Results were expressed as frequencies or as mean ± SD unless indicated otherwise. The χ2-test was used to compare proportions, and Student t test was used to compare means. The significance level of all analyses was set to 5%, and exact p values are reported. Results were expressed using descriptive statistics.

## Results

**Figure 2:**
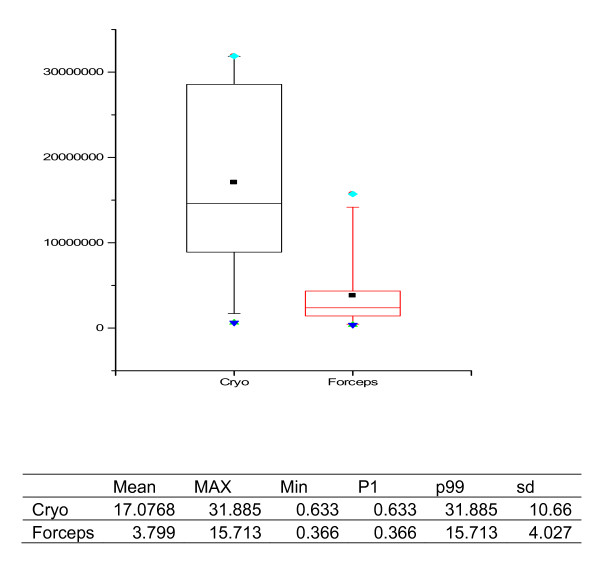
**Specimen Size, graph in μm^2^, statistical data in mm^2^**.

**Figure 3:**
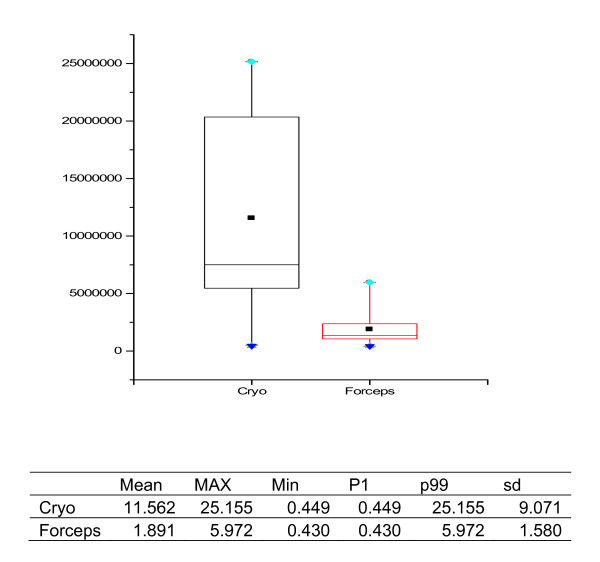
**Size of the alveolar part, graph in μm^2^, statistical data in mm^2^**.

Morphometric data of all samples including presence of alveolar part, its size and artefacts within alveolar tissue are listed in tables 1 and 2, and representive specimens retrieved with the two methods under comparison are illustrated in Figure 2 and 3.

Cryoprobes were larger and much more representative of real lung structure featuring pathological attributes as compared to small biopsy specimens of the forceps technique (Figure 1). Specimen size in cryobiopsies was significantly larger than in those obtained by forceps (mean: 17.1 ± 10.7 μm^2 ^versus 3.8 ± 4.0 μm^2^, n = 15 and 18 respectively; p < 0.001) (Figure 2). In cryobiopsies, alveolar tissue was found in 11 of 15 (73%), in forceps biopsies only in 10 of 18 (56%), thus showing a trend for cryobiopsy containing alveolar tissue more often (p = 0.290). The specimens lacking alveolar tissue either contained only bronchial mucosa and cartilage or proved to be flat long bands of inner bronchial wall lining, the latter phenomenon exclusively seen in cryoprobes. In specimens with alveolar tissue, its size was significantly larger in the cryobiopsies (11.6 ± 9.1 μm^2 ^versus 1.9 ± 1.6 μm^2 ^in the forceps group, n = 11 and 10 respectively; p = 0.004) (Figure 3). When the pathologists rated the presence of artefacts within the alveolar tissues, none of the specimens taken by cryobiopsy technique (0/11; 0%) showed parenchymal damage due to compression, whereas all (10/10; 100%; p = 0.005) forceps samples demonstrated at least mild parenchymal changes.

In both groups of patients there were no complication in terms of pneumothorax. In one patient of the forceps biopsy group a major bleeding (> 3 min) occurred without need of further intervention.

## Discussion

Transbronchial forceps biopsy is a well accepted and established retrieval tool for the histologic analysis of parenchymal lung disease [1]. The requirement of histologically proven diagnosis in suspected lung cancer is evident. However, on the background of tumor heterogeneity, forceps biopsies are always hampered by small sample sizes and thus uncertain representation for the tumor. In addition, larger samples will likely become even more important regarding the steadily increasing amount of information that is expected to be gathered from tissue samples in pinhead size. Particularly surface and receptor analyses, prognostic or predictive genetic markers and/or upcoming epigenetic changes will become more important.

In non-neoplastic diseases a number of entities like sarcoidosis or bronchiolitis obliterans may be diagnosed on material obtained by transbronchial forceps biopsy. Thus far, the efficacy of TBB in non-neoplastic lung diseases has only been addressed in a few studies [1,4,10,12].

Small size is the major factor limiting the usefulness of TBB in clinical practice. Therefore the adequacy of samples has always been a matter of debate. In needle biopsies of solid organs such as kidney or liver with histologically distinct and thus countable structures (e.g., number of glomeruli), specimen adequacy can be determined easily. In contrast, specimen adequacy in lung biopsy has not yet been clearly defined and depends on clinical context. However no such approach has been implemented, because alveoli vary largely in size, shape and quality and cannot be easily rated. Moreover, two separate histologic structures - bronchioli and alveoli - should be present in a biopsy. Additionally there are several artifacts, which are typical for a forceps biopsy such as atelectasis, intraalveolar hemorrhage and so called bubble artifacts [4]. The frequent presence of artificial atelectasis may obscure diagnostic features and also be misinterpreted as interstitial fibrosis [10]. Several studies have rated the adequacy of the transbronchial biopsy based on alveolar content and specimen size. In a multivariate analysis, the number of alveolar spaces necessary for an adequate biopsy was defined as 20 [6]. Morphometry has been shown to be an efficient method to evaluate sample size [6].

A promising approach to obtain more representative samples in bronchial biopsy seems to be the introduction of modern cryoprobes. With this technique, the sample is collected while still being frozen, with the tissue attached on the frozen probe's tip. The value of the biopsy under diagnostic aspects is influenced not only by the size itself but by the absolute as well as relative content of alveolar structures, bronchial wall and neoplastic or reactive changes of the tissue samples [1,2,6,7,9,12,18].

In our series the size of the biopsy specimens differed significantly between cryoprobes and TBB specimens. However it has to be emphasized that in each TBB only one of the up to 4 biopsies was included in the analysis. Usually we decided to include the largest piece. In cases lacking alveolar tissue, present in other biopsies of the case, we included the largest alveoli-bearing biopsy specimen. The rationale behind this was the fact that for the diagnostic value of a biopsy only continuous tissue areas can be included in the diagnostic process, as topographic information is essential in histopathology, especially in diagnoses predominantly driven by pattern information. Strictly speaking every specimen in TBB has to be interpreted for its own, as the exact topographic relation between specimens of different biopsy site remains unclear, at least for the histopathologist.

Our results show an important difference between forceps biopsy and cryobiopsy. The latter samples are larger, and a trend to superiority with respect to the alveolar tissue fraction is observable. In the group of cryobiopsies we found alveolar tissue in a higher proportion of cases as compared to the group of forceps biopsies (73 vs 56%). The absolute value as well as the relative amount of alveoli is undoubtedly superior in the cryoprobe. In addition, artifacts in the alveolar part were not observed in the cryogroup but in each sample obtained by forceps biopsy.

The increased amount of tissue available for histological and molecular access may significantly improve the diagnostic value of bronchoscopical lung biopsies which has to be investigated in further studies.

## Conclusions

Major findings of this study were: 1. Cryobiopsy specimens are significantly larger than those obtained by forceps transbronchial biopsies. 2. There was a tendency for alveolar tissue to be recovered more likely using cryobiopsies. 3. The size of the alveolar part in cryobiopsy specimens was significantly larger compared to specimens obtained by forceps. 4. Cryobiopsies appear of higher quality to the pathologist lacking more or less any artifacts in the alveolar parts of specimens. Cryotechnique is an important new tool for the bronchoscopic diagnosis of lung disease. The method is superior to TBB with a forceps in terms of sample size and quality. Whether this technique can replace even open surgical biopsy for the diagnosis of parenchymal lung disease needs to be addressed by prospective studies.

## List of abbreviations

TBB: Transbronchal biopsy; OL: Lung upper lobe; ML: Lung middle lobe; UL: Lung lower lobe; ILD: Interstitial lung disease

## Competing interests

The authors declare that they have no competing interests.

## Authors' contributions

WA, NS, WG, JK collected and registered all the samples, SG and TM performed histological examinations and measurements on samples, statistical analysis was performed by TTB and TM, patient and clinical data documentation was done by TB, JK and WG, endoscopical implementation and processing of cryotechnique: WG and NS, manuscript: SG and WG, study design: TM and WG, study coordination: TM and WG.
