# Erratum to: Morphometrical analysis of transbronchial cryobiopsies

**DOI:** 10.1186/s13000-016-0515-1

**Published:** 2016-07-19

**Authors:** Sergej Griff, Wim Ammenwerth, Nicolas Schönfeld, Torsten T. Bauer, Thomas Mairinger, Torsten-Gerriet Blum, Jens Kollmeier, Wolfram Grüning

**Affiliations:** Institute of Pathology, HELIOS Klinikum Emil von Behring, Berlin, Germany; Department of Pneumology, Lungenklinik Heckeshorn, HELIOS Klinikum Emil von Behring, Berlin, Germany

## Erratum

The original version of this article [1] was unfortunately missing an acknowledgments section.

The information missing from this section should have noted that this work is a part of the doctoral thesis of main author Sergej Griff, being performed at the Charité, University of Medicine in Berlin, Germany.

## References

[1] Griff S, et al. Diagnostic Pathology 2011, 6:53 DOI: 10.1186/1746-1596-6-53. http://www.diagnosticpathology.org/content/6/1/53. 10.1186/1746-1596-6-53 PMC3127816 21679402

